# Pulmonary alveolar proteinosis complicated by lung cancer with favorable prognosis: a case report and literature review

**DOI:** 10.3389/fonc.2024.1434631

**Published:** 2024-09-12

**Authors:** Ying Wu, Wenhui Guan, Jiaxi Deng, Wenwei Mo, Beini Xu, Jiahao Zhang, Huixin Jiang, Jie Liu, Xinqing Lin, Chengzhi Zhou

**Affiliations:** ^1^ State Key Laboratory of Respiratory Disease, National Clinical Research Center for Respiratory Disease, National Center for Respiratory Medicine, Department of Respiratory and Critical Care Medicine, Guangzhou Institute of Respiratory Health, the First Affiliated Hospital of Guangzhou Medical University, Guangzhou, Guangdong, China; ^2^ Graduate School, Guangzhou Medical University, Guangzhou, China; ^3^ Graduate School, Sun Yat-sen University, Guangzhou, China

**Keywords:** lung cancer, pulmonary alveolar proteinosis, checkpoint inhibitor pneumonitis, granulocyte-macrophage colony-stimulating factor, immunotherapy

## Abstract

With the increasing incidence of lung cancer, the coexistence of pulmonary alveolar proteinosis (PAP) and lung cancer is becoming more common. However, the standard treatment protocols for patients with both conditions are still being explored. The conflict between the rapidly evolving therapeutic approaches for tumors and the limited treatment options for PAP presents a significant challenge for clinicians. Determining the optimal timing of treatment for both conditions to maximize patient benefit is a clinical conundrum. Here, we report a rare case of PAP complicated by lung adenocarcinoma, where interstitial lung changes worsened after neoadjuvant therapy but improved significantly following surgical resection of the lung adenocarcinoma. This case highlights the importance of prioritizing tumor treatment in patients with lung cancer complicated by PAP and examines the interplay between the two conditions, as well as potential therapeutic strategies.

## Introduction

Pulmonary alveolar proteinosis (PAP) is a rare diffuse interstitial lung disease characterized by abnormal accumulation of surfactant in alveolar macrophages and alveolar spaces. Clinically, it presents with dyspnea and increases the risk of secondary infections and/or fibrosis. PAP can be categorized into primary PAP, secondary PAP, and congenital PAP based on the etiology. Primary PAP results from disrupted granulocyte-macrophage colony-stimulating factor (GM-CSF) signaling and can be classified as autoimmune PAP (due to GM-CSF autoantibodies) or hereditary PAP (due to mutations in genes encoding GM-CSF receptor subunits). Secondary PAP arises from reduced function and/or number of alveolar macrophages due to various causes, including malignant and non-malignant hematologic disorders, non-hematologic malignancies, immunodeficiency syndromes, chronic inflammatory syndromes, and chronic infections. Congenital PAP results from gene mutations causing surfactant production disorders and is commonly seen in children. Currently, there is no standardized treatment for PAP. For symptomatic PAP, the recommended therapeutic options include whole-lung lavage (WLL), emerging therapies based on pathogenesis (e.g., recombinant GM-CSF therapy, Rituximab, plasma exchange), and lung transplantation (1).

Lung cancer is a leading cause of cancer-related mortality worldwide, with an increasing incidence each year. Reports of PAP complicated by lung cancer are also on the rise. Although treatment modalities for lung cancer have become increasingly diverse with advances in oncology, there is still no standard therapy for lung cancer complicated by PAP. Here, we report a rare case of autoimmune PAP (aPAP) complicated by lung adenocarcinoma, where interstitial lung disease worsened after neoadjuvant therapy, but PAP improved significantly following surgical resection of the lung adenocarcinoma.

## Case Presentation

**Figure 1:**
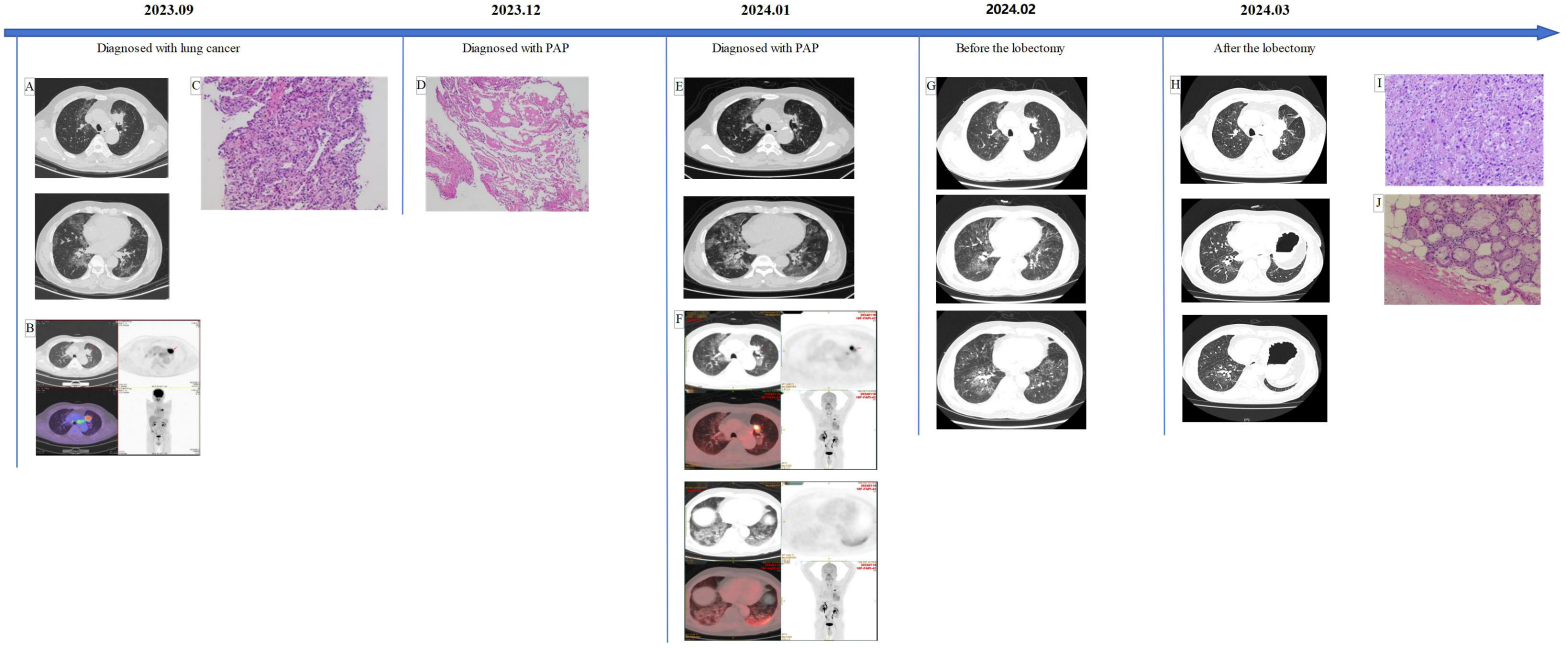
**(A, B)** An active-metabolism solid mass in the upper lobe of the left lung with lymph nodes in the mediastinum and left hilum, as well as interstitial inflammation in both lungs. **(C)** The atypical cells are arranged in the shape of small nests or glands, and the nucleus is enlarged and deeply stained. **(D)** A small amount of eosin granules can be seen, special staining results: PAS-D (+), PAS (+). **(E, F)** The nodules became smaller than at the first visit, while the multifocal honeycomb consolidation shadows of both lungs were larger. **(G)** The tumor was slightly larger than when diagnosed with PAP, and the imaging changes of PAP decreased. **(H)** Postoperative changes in the left lung, ground glass changes in the lungs were significantly reduced. **(I, J)** Alveolar and complex glandular growth patterns can be seen, with a small amount of adherent growth, fibrous tissue hyperplasia in some areas, scattered and focal lymphocyte infiltration with foam cell aggregation, and focal necrosis. Combined with the clinical history, it is consistent with pulmonary invasive adenocarcinoma. After neoadjuvant treatment, the primary tumor showed partial relief and changes; no cancer was found in the bronchial tissue (stump).

A 60-year-old male patient with a 80 pack-years smoking history experienced cough, expectoration, and exercise-induced shortness of breath for one month, as well as sudden chest tightness for one day. He was diagnosed with lung cancer and bilateral interstitial pneumonia at a local hospital. Positron emission tomography/computed tomography (PET/CT) revealed an active-metabolism solid mass in the upper lobe of the left lung, with lymph nodes in the mediastinum and left hilum, as well as interstitial inflammation in both lungs (**Figure 1**). Endobronchial biopsy (EBB) of the mediastinal lymph nodes (groups 7 and 4L) did not detect significant tumor cells. Histopathological examination of a percutaneous transthoracic lung biopsy (PTLB) revealed adenocarcinoma (programmed cell death 1 ligand 1, PD-L1 95%; tumor mutation burden, TMB 9.4). Therefore, the patient underwent two cycles of neoadjuvant therapy (Tislelizumab plus Carboplatin and Pemetrexed plus Bevacizumab) and subsequently underwent a chest CT scan to evaluate for surgery. While the nodule in the upper lobe of the left lung reduced in size, interstitial inflammation of both lungs persisted.

The patient then presented to our hospital with complaints of cough, expectoration, and dyspnea after speaking for a while. His physical examination was unremarkable, and he had no history of exposure to occupational or environmental fibrosis. Laboratory tests revealed: lactate dehydrogenase 179.5U/L, carcinoembryonic antigen 9.72ng/mL, cytokeratin 19 fragment 7.09ng/mL, interleukin-6 1259.46 pg/mL, interleukin-8 60.70 pg/mL, interleukin-10 21.66 pg/mL. Chest CT reexamination showed similar findings to the prior scan at the other hospital. Before receiving the results of the EBB biopsy, we treated the patient empirically with hormone therapy, antifibrotic medication (Pirfenidone administered three times daily, starting at 300mg and gradually increased to 600mg), and human immunoglobulin. EBB of left lung revealed positive Periodic Acid-Schiff (PAS) with diastase and PAS staining, suggesting of alveolar proteinosis (**Figure 1**). [18F]FAPI-42 PET/CT indicated a reduction in nodules in the left lung but an increase in multifocal honeycomb consolidation shadows in both lungs, with significant fibrotic changes in the lower left lung (**Figure 1**). While awaiting surgery, we continued previous hormone and antifibrotic therapies and increased spleen aminopeptide to regulate immunity. After one month, the patient underwent thoracoscopic left upper lobectomy and lymph node dissection, with final staging of ypT3N0M0. Surprisingly, Preoperative chest CT scans showed a reduction in ground-glass opacities in the lungs (**Figure 1**). Ten days post-surgery, a chest CT scan showed further improvement in ground-glass opacities (**Figure 1**). Meanwhile, laboratory tests reported a serum GM-CSF antibody level of 15.94268014 μg/ml. The one-month follow-up showed that the patient has recovered well and is symptom-free.

## Discussion

Currently, there have been only around 10 reported cases worldwide of solid tumors, including lung cancer which accounts for about 70% of the cases retrieved, in conjunction with PAP (**Table 1**). And instances of primary lung cancer combined with PAP typically involve PAP being diagnosed prior to lung cancer or both conditions being identified concurrently (2–17) (**Table 1**). Limited information is available regarding treatment approaches and prognosis for this comorbid condition, with 7 of the 17 patients retrieved explicitly reporting improvements in PAP following tumor therapy. In this paper, we describe a case of PAP co-occurring with primary lung cancer that resulted in a favorable prognosis.

**Table 1:** Summary of previous cases of PAP combined with solid tumors.

Author	Year	Gender	Age	Smoking History	Cancer	Stage	Preceding Diseases	PAP Type	Tumor therapy	PAP therapy	Whether PAP improved after tumor treatment
Kadota J	1999	Male	67	YES	LUSC	T1N0M0	unknown	unknown	Surgical excision	WLL	unknown
Kim K-H	2004	Male	59	——	LUSC	T2N1M0	unknown	unknown	Surgical excision	WLL	unknown
Cheewakriangkrai R	2005	Male	45	NO	MESO	——	PAP	unknown	Chemotherapy	WLL	unknown
Su K-C	2007	Female	54	NO	LUAD	T2N2M0	PAP	aPAP	Surgical excision	——	NO
Su K-C	2007	Female	59	NO	LUAD	——	unknown	unknown	No treatment	No treatment	unknown
Figueiredo AA	2007	Male	54	——	ERMS	——	unknown	unknown	Surgical excision	NO	NO
Liu H	2014	Male	58	YES	LUSC	T1N0M0	PAP	Secondary PAP	Surgical excision	NO	YES
Sawai T	2014	Female	48	NO	BRCA	——	unknown	aPAP	Surgical excision	NO	YES
Iwakami S	2015	Female	78	NO	LUAD	——	PAP	aPAP	EGFR-TKI	WLL	unknown
Zhou W	2015	Male	42	YES	KIRC	T1aN0M0	unknown	Secondary PAP	Surgical excision	NO	YES
Hiraki T	2015	Male	50	YES	SCLC	——	PAP	——	unknown	NO	Death
Zhang N	2018	Male	47	——	LUAD	T1cN3M0	unknown	Tend to secondary PAP	Surgical excision+Adjuvant chemotherapy	NO	YES
Shimizu N	2021	Male	61	——	KIRC	——	PAP	aPAP	Surgical excision	NO	NO
Shimoda M	2023	Female	46	——	Combined LUAD and LUSC	T4N3M1a	Tumor	aPAP	First line: EGFR-TKI, Second- and third-line: chemotherapy, Fourth-line: Docetaxel and Ramucirumab	NO	YES
Xiang Y	2023	Male	55	——	SCLC	LS-SCLC	PAP	aPAP	Chemotherapy+Sequential radiotherapy	NO	YES
Papaioannou O	2023	Male	60	YES	SCLC	LS-SCLC	unknown	aPAP	Chemotherapy	WLL+Rituximab+corticosteroid +plasma exchange+inhalation Salbutine maintenance therapy	YES
Horie M	2023	Male	62	——	LUAD	T2aN0M1c	PAP	aPAP	Chemotherapy	Left lung lavage	unknown

squamous cell carcinoma, LUSC; mesothelioma, MESO; adenosquamous carcinoma, LUAD; prostate embryonal rhabdomyosarcoma, ERMS; breast cancer, BRCA; kidney clear cell carcinoma, KIRC; limited- stagesmall cell lung cancer, LS-SCLC; pulmonary alveolar proteinosis, PAP; autoimmune PAP, aPAP; Epidermal growth factor receptor tyrosine kinase inhibitor, EGFR-TKI; Whole-lung lavage, WLL.

In this case, interstitial changes were observed in both lungs when a space-occupying lesion was noted in the patient’s lungs. After two courses of neoadjuvant immunotherapy, the tumor responded positively, but the interstitial changes in both lungs were still existed. Following empirical administration of corticosteroids, antifibrotic agents, and immune-modulating therapy, a re-lung biopsy indicated that the interstitial changes were pathologically consistent with PAP and re-chest CT indicated progression of interstitial changes in both lungs. At this point, we considered the possibility that the patient had lung cancer in combination with PAP, with potential progression of PAP post-neoadjuvant immunotherapy, or checkpoint inhibitor pneumonitis (CIP). Consequently, we discontinued neoadjuvant immunotherapy and proceeded with an elective lobectomy following hormonal, antifibrotic, and immune-modulating treatments. Preoperative chest CT scans showed that the PAP lesions had resolved, and the patient’s shortness of breath improved. Moreover, the patient’s symptoms completely resolved after lung cancer resection, and chest CT scans revealed a significant remission in PAP lesions in both lungs, suggesting that the tumor might have caused secondary PAP. However, at this point, the presence of high GM-CSF antibody levels in the patient’s serum contradicted this speculation.

In several previously reported cases, improvement in symptoms and imaging related to PAP was observed following treatment focused solely on the tumor. However, it remains uncertain whether publication bias exists in these reports (7, 8, 14–16). It is undeniable that PAP combined with primary lung cancer cannot be understood as two completely independent diseases, nor can it be explained by a separate causal theory. Further investigation is needed to understand the connection between them. Patients with interstitial lung disease (ILD) have an increased risk of lung cancer compared to those without underlying lung disease (18). In both ILDs and lung cancer, dysregulation of kinases and phosphatases can lead to aberrant activation of growth-promoting pathways (such as phosphoinositide 3-kinase (PI3K) - protein kinase B (Akt) and mitogen-activated protein kinase (MAPK) signaling pathways) or inactivation of growth-inhibitory pathways (such as transforming growth factor beta (TGF-β) signaling), promoting uncontrolled cell proliferation and survival, contributing to the pathogenesis of both diseases (19). Further studies have found that idiopathic pulmonary fibrosis (IPF) and lung adenocarcinoma (LUAD) share key signaling pathways, including the PI3K-Akt pathway. Epithelial cells have been identified as specific cell types involved in the progression of IPF to LUAD comorbidity (20). Mascaux et al. identified dysregulation of various extracellular signals in ILDs and lung cancer, including growth factors (e.g., TGF-β, platelet-derived growth factor (PDGF)), cytokines (e.g., interleukin-6 (IL-6), tumor necrosis factor alpha (TNF-α)), and extracellular matrix components (e.g., collagen, fibronectin). Dysfunctional signaling pathways disrupt normal tissue repair mechanisms in ILDs and promote the proliferation, invasion, and metastasis of tumor cells in lung cancer (21). Furthermore, Vancheri noted that dysregulation of apoptotic pathways can impair cell death mechanisms, allowing abnormal cells to survive and proliferate. This resistance to apoptosis promotes tissue remodeling in ILDs and facilitates tumor progression in lung cancer (22). Reduced intercellular communication further disrupts normal cell-cell interactions, promoting tissue remodeling in ILDs and tumor progression in lung cancer (23). Previous studies have demonstrated that methylation of tumor suppressor genes and/or hypomethylation of oncogenes play a critical role in cancer development, and these epigenetic changes also occur in IPF. Approximately 10% of mRNA is aberrantly expressed in IPF, associated with genes involved in the fibrosis-related genomics, capable of influencing epithelial-mesenchymal transition (EMT) induction, cell apoptosis, and extracellular matrix regulation which are related to cancer biology (23). Despite these findings, as a special type of interstitial lung disease, the causal relationship and interaction mechanism between PAP and lung cancer remain unclear.

On one hand, previous studies have noted alveolar proteinosis (AP)-like changes in tissues adjacent to non-small cell lung cancer, with type II alveolar cells and neutrophils playing crucial roles in mediating AP morphological changes (24, 25). At the same time, certain immunosuppressive chemicals secreted by lung cancer cells may inhibit macrophages locally and potentially induce PAP (20, 21). On the other hand, Friemann’s animal experiments demonstrated dose- and time-dependent patterns of type II alveolar epithelial cell proliferation in rats after the induction of alveolar proteinosis. The increase in mitosis of type II alveolar epithelial cells and the heightened alveolar proteinosis and proliferative activity of alveolar cells could potentially promote tumor development (26). The expression of GM-CSFR and the secretion of GM-CSF can inhibit the proliferation of malignant cells by inducing G0/G1 phase arrest in the cell cycle and enhancing cell differentiation to directly inhibit tumor growth. In a small cell lung cancer cell line, the addition of anti-GM-CSF antibody abolished the antiproliferative effect of GM-CSF (27). The role of GM-CSF in lung cancer combined with PAP warrants further investigation. In summary, there is no further research on the mechanism of the occurrence and development of lung cancer and PAP recently, and we can only find that there is a relationship between them from the existing literatures, which poses challenges to clinical treatment.

Currently, there is no established standard treatment plan for PAP. The recommended treatment options for PAP with clinical symptoms mainly include WLL, emerging therapies based on pathogenesis (such as recombinant human GM-CSF therapy, rituximab, plasma exchange, etc.), and lung transplantation (1). WLL has been utilized in several cases of PAP complicated by lung cancer, providing a degree of relief from respiratory symptoms and lung opacities while allowing more time for tumor treatment (2, 3, 16, 17). Another study found that WLL helped maintain stable arterial oxygen levels in patients with lung cancer-associated bronchial obstruction under cutaneous veno-venous extracorporeal membrane oxygenation (ECMO), which may reduce the risk of fatal hypoxia (3). Recombinant human GM-CSF (rhu GM-CSF), known for its innate and adaptive immune activity, is thought to improve cancer prognosis and reduce the toxicity of chemotherapy and other immunotherapies (28). However, its effectiveness as a treatment for aPAP in patients with tumors has not yet been explored. Nevertheless, there is evidence that GM-CSF can mediate the overexpression of PD-L1 on tumor-associated macrophages (TAMs) in the tumor immune microenvironment through the GM-CSF/STAT5 signaling pathway, contributing to tumor immune escape mechanisms. Additionally, high GM-CSF levels are associated with poor outcomes in cancer patients (29, 30). Therefore, the use of rhu GM-CSF should be approached with caution.

It was previously thought that radiotherapy could stimulate the production of surfactant in type II alveolar cells and impair the function of macrophages. However, in a case of small cell lung cancer with PAP treated with chemotherapy followed by thoracic radiation therapy, PAP completely disappeared. Further experiments in mice suggested that radiation may induce self-renewal of alveolar macrophages, aiding in restoration of normal pulmonary surfactant clearance (15). Certain commonly used cancer drugs may also contribute to the development of PAP. For instance, it has been reported that tyrosine kinase inhibitors (TKIs) can regulate the GM-CSF signaling pathway in monocyte-macrophage lineage cells. Additionally, immunosuppressive agents, chemotherapy, and corticosteroids may exacerbate PAP by inhibiting the phagocytosis and catabolism of alveolar macrophages (31, 32). Shimoda M reported a suspicious case of increased PAP caused by Osimertinib therapy for lung cancer (14). Simultaneously, Horie M reported a case of safe use of immune checkpoint inhibitors (ICIs) - Atezolizumab in lung cancer patients with PAP, and there were no previous reports of PAP progression or occurrence of CIP after ICIs treatment (17). However, in this particular case, the patient experienced increased shortness of breath and fibrosis after two courses of neoadjuvant immunotherapy. Whether this was due to aPAP deterioration caused by cancer treatment or CIP remains unclear. Moreover, the real-world incidence of CIP in lung cancer patients is not low, so the safety of immunotherapy in the treatment of lung cancer patients with PAP requires further investigation.

Immunomodulatory therapy refers to treatments that modify or regulate the immune system’s response which can modulate an inadequate or amplified immune response to reduce the clinical course of the disease and restore homeostasis (33). As previously mentioned, one of the characteristics of PAP is abnormal deposition of protein in the alveoli, primarily due to impaired function of alveolar macrophages in clearing these proteins (2). Immunomodulatory therapy can enhance or activate the function of alveolar macrophages, helping them to more effectively clear proteins from the alveoli, thereby reducing or halting the progression of deposition. PAP may be associated with autoimmune reactions, and immunomodulatory therapy can help suppress inappropriate immune responses, reducing the release of immune mediators and thus minimizing alveolar damage and protein deposition (2, 34). Additionally, due to impaired lung function and abnormal immune system in PAP patients, they are prone to complications such as secondary infections (35). Immunomodulatory therapy can help regulate immune balance and reduce the risk of infections. Overall, immunomodulatory therapy in PAP could repair or improve immune system function through various mechanisms, directly or indirectly reducing abnormal protein deposition in the alveoli, thereby improving lung function, preventing complications, and enhancing long-term quality of life and prognosis for patients. Regarding this case patient, in addition to the classic immunomodulatory drug-immunoglobulin, spleen aminopeptide lyophilized powder therapy was also administered in the later stage. Spleen aminopeptide primarily consists of polypeptides and nucleotides derived from fresh pig spleen, containing diverse amino acids and immunomodulatory factors (36). Several clinical studies have shown spleen aminopeptide therapy plays an important role in the adjuvant therapy of tumors, organ injuries, immune disorders and infections. Wu et al. found that adding spleen aminopeptide oral lyophilized powder (SAOLP) to standard therapy enhances immune function and the ability to resist human cytomegalovirus (HCMV) infection in infants and children, significantly reducing liver damage (36). Compared to aerosol inhalation therapy alone, combined spleen aminopeptide inhalation therapy demonstrates significantly improved efficacy in treating pediatric pneumonia (37). As a convenient and safe adjuvant therapy, SAOLP can improve the Health-Related Quality of Life (HRQoL) and immune response in advanced cancer patients, promoting anti-tumor immunity (35). In cases where cancer and autoimmune diseases coexist, immunomodulatory therapy can act as adjunctive treatment to alleviate disease progression and regulate the autoimmune response, thus aiding subsequent therapies and promoting a favorable prognosis. However, specific treatment protocols still necessitate further consideration by clinical physicians and additional support from clinical and basic research for validation.

## Conclusion

The relationship between PAP and lung cancer is complex, involving pathogenesis and treatment. PAP may improve after chemotherapy, radiotherapy, and immunotherapy for tumors; however, it is unclear whether this increases the risk of potential complications from cancer treatment. There is a pressing need for further research into the treatment mechanisms and safety for patients with both conditions.

## Data Availability

The original contributions presented in the study are included in the article/supplementary material. Further inquiries can be directed to the corresponding author/s.
